# A Personalized and Smart Flowerpot Enabled by 3D Printing and Cloud Technology for Ornamental Horticulture

**DOI:** 10.3390/s23136116

**Published:** 2023-07-03

**Authors:** Yecheng Li, Jiaxing Luo, Zixuan Liu, Daosheng Wu, Cheng Zhang

**Affiliations:** College of Engineering, Nanjing Agricultural University, Nanjing 210031, China; liyecheng@stu.njau.edu.cn (Y.L.); 9193012025@njau.edu.cn (J.L.); 9213011016@stu.njau.edu.cn (Z.L.); 2022812079@stu.njau.edu.cn (D.W.)

**Keywords:** sensors, smart flowerpot, automatic control, real-time monitor

## Abstract

This paper presents a personalized and smart flowerpot for ornamental horticulture, integrating 3D printing and cloud technology to address existing design limitations and enable real-time monitoring of environmental parameters in plant cultivation. While 3D printing and cloud technology have seen widespread adoption across industries, their combined application in agriculture, particularly in ornamental horticulture, remains relatively unexplored. To bridge this gap, we developed a flowerpot that maximizes space utilization, simplicity, personalization, and aesthetic appeal. The shell was fabricated using fused deposition modeling (FDM) in 3D printing, and an Arduino-based control framework with sensors was implemented to monitor critical growth factors such as soil moisture, temperature, humidity, and light intensity. Real-time data are transmitted to the Bamfa Cloud through Wi-Fi, and a mobile application provides users with instant access to data and control over watering and lighting adjustments. Our results demonstrate the effectiveness of the smart flowerpot in enabling automated monitoring of plant growth and environmental control. This innovation holds significant promise for advancing smart device development in ornamental horticulture and other related fields, enhancing efficiency, plant health, and overall user experience. Future research in this area has the potential to revolutionize horticultural practices and contribute to the advancement of smart agriculture.

## 1. Introduction

Agricultural development is significantly promoted by advanced technologies [1]. Take molecular breeding as an example. As one of the main innovations in plant breeding, it detects specific molecular characteristics or introduces specific genes to make the selection of target traits [2]. At present, it mainly contains marker-assisted selection and transgenic breeding [3,4]. Compared with conventional breeding, it is more effective, accurate, and free from the interference of environmental conditions [5]. In addition, it allows people to produce new varieties which meet public requirements. Another example is agricultural remote sensing. It depends on the satellite-based sensors and sensors on manned or unmanned planes detecting electromagnetic waves that items continuously reflect and absorb, so to obtain the details of the plants [6]. Compared to agricultural surveying techniques, it provides an opportunity for farmers and researchers to assess the plants’ condition through visual materials acquired by remote sensing rather than touching them physically [7,8]. Consequently, these innovative technologies have induced a paradigm shift in agricultural practices, steering the sector towards increased efficiency and intelligence.

Three-dimensional (3D) printing technology, characterized by its additive manufacturing process, is capable of fabricating physical objects from digital geometric data [9]. This process enables the production of complex shapes in mass customization scenarios, eliminating the need for traditional molding methods [10]. Currently, 3D printing has been widely adopted across various sectors, including aerospace [11], automotive industry [12], medical and healthcare industry [13,14,15], architecture and construction industry [16,17], fabric and fashion industry [18,19], electric and electronic industry [20,21], etc. The technology’s inherent versatility and capacity for personalization underpin its applicability across these diverse domains [22]. In parallel, cloud technology, a form of hosting technology, integrates hardware, software, and network resources over a wide area network or local area network to facilitate data computation, storage, processing, and sharing [23]. Its application spectrum extends to finance [24], manufacturing [25], education [26,27], medicine [28,29], and data storage [30], among others. The technology enables users to store data and dispatch instructions to their smart devices. Despite the widespread applications of these two technologies, their combined utilization in the agricultural sector remains relatively unexplored.

Ornamental horticulture, a specialized discipline within the broader field of agriculture, encompasses the cultivation of a vast and diverse array of plants or plant parts primarily for aesthetic purposes [31,32]. By enhancing the beauty of living, working, and recreational spaces, ornamental horticulture contributes significantly to environmental aesthetics and the enhancement of human physical and mental well-being. Moreover, it constitutes a crucial economic segment within horticulture [33]. Ornamental horticulture contributed $348 billion output totally to the US economy in 2019, and the industry’s continuous expansion has yielded substantial economic benefits [34]. A critical component within this sector is the flowerpot, which plays an integral role in the cultivation and propagation of ornamental plants [35]. Flowerpots are difunctional: they provide both an optimal environment for plant growth and an intrinsic ornamental value that aligns with user aesthetics.

**Figure 1:**
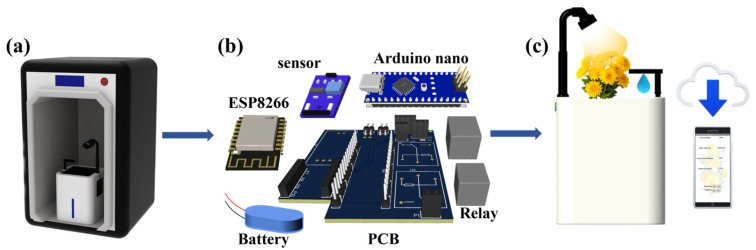
Manufacturing process, concept, and application of personalized and smart flowerpot. (**a**) 3D printing of the shell of the personalized and smart flowerpot. (**b**) The structure of the integrated circuit board. (**c**) Conceptual graph of the multi-sensor-based smart flowerpot which contains the data on soil moisture, light intensity, and supplemental light and watering according to plant needs and the mobile application that displays the real-time data from the cloud technique.

Herein, we developed a personalized and smart flowerpot enabled by 3D printing and cloud technology for ornamental horticulture to break through the limitations of existing designs (Table S1 [36,37,38,39,40,41]). Its shell design mainly considered maximum space utilization, simplicity, alignment with the user’s aesthetics, and most importantly, personalization. Its shell was first fabricated by FDM, which is a type of 3D printing (Figure 1a and Figure S1). Afterwards, an Arduino-based control framework equipped with a suite of sensors that monitor critical growth factors was assembled with control and acquisition circuitry based on low energy consumption (Figure 1b). The factors that the suite of sensors can detect include soil moisture, temperature, humidity, and light intensity. The integrated circuit board was installed into the 3D printed personalized shell. Due to the low power consumption of the electronic components and Wi-Fi data transmission, a rechargeable lithium-ion battery (5 V 5600 mAh) was selected and tested for meeting user requirements for over 7 days. To facilitate uninterrupted data transmission and monitoring, the Arduino processes sensor data and transfers them to the Bamfa Cloud via Wi-Fi. A mobile application was developed to display the real-time data from the Bamfa Cloud. The system features a multifunctional printed circuit board (PCB) integrating multiple circuits, consolidating several functionalities into one compact platform. The accompanying mobile application allows users to scrutinize plant growth conditions and control the system’s operations, such as watering and lighting adjustments (Figure 1c).

## 2. Experimental Section

### 2.1. 3D Printing of the Shell

The structure of the shell was designed and modeled in CAD software (Creo, Durham, NC, USA) and rendered using KeyShot. It needs to be assembled after printing out various parts due to its medium to large entity. Compared to other layered manufacturing technologies, FDM technology can produce high-quality parts by adjusting different azimuth angles and layer thicknesses and by calculating the minimum volumetric error through computer-aided design (CAD) [42,43]. Our smart flowerpot is able to water and fill light as needed, thus promoting sustainability by minimizing water waste and reducing electricity usage. As a thermoplastic, polylactic acid (PLA) is derived from 100% renewable resources. Its sources include sugar, corn, potatoes, sugarcane, sugar beets, and so on. When PLA materials are disposed of, they undergo degradation, contributing to their eco-friendliness and overall sustainability [44]. In addition, PLA materials applied in horticulture exhibit renewability, sustainability, and biocompatibility. In terms of production, PLA shows advantages of low energy consumption. Notably, PLA material employed for 3D printing yields a shell with commendable strength and hardness properties [45,46]. The outer shell of the flowerpot adopts the Liantai Fortus 450 mc high-precision engineering plastic production equipment, and the Fortus system is based on Stratasys FDM technology. By using FDM technology, a type of material extrusion, the 3D printer extrudes PLA thermoplastic filaments. At the same time, it controls the temperature and the flow rate of the polymer accurately. PLA melts at the spray nozzle and remains in a continuous semi-liquid state. The melted PLA filaments are extruded and placed on a platform layer after layer. On the platform, PLA filaments chill, solidify, and attach to the previously deposited material. The printing speed of FDM is generally controlled at 45 mm/s, and since the material used is PLA, the temperature is generally controlled at 175–195 °C [47] and the layer thickness is 0.1 mm. The 3D printer’s spray nozzle or the platform moves along the xy level and the molten materials are placed on the plate. In addition, the platform or the nozzle moves vertically (z-direction), layer by layer, until all layers are finished and solidify into a completed 3D flowerpot. Then, a 2 mm assembly gap is reserved for each part to facilitate the assembly of the flowerpot’s body. After 3D printing, the protective shell is polished, and the corresponding design components are colored accordingly. As for the life of this shell made of PLA, after being exposed to temperatures of 50–60 °C for approximately 45–60 days within a composting environment, PLA undergoes hydrolysis, leading to the formation of smaller molecules such as oligomers, dimers, and monomers [44]. However, after the shell of the flowerpot was printed out, it was painted to provide some protection. Secondly, our flowerpot only has a detachable inner liner that comes into contact with the soil and water, and the temperature during use cannot reach 50–60 °C, so the lifespan of the flowerpot is much longer than 45–60 days. In addition, even if the inner liner is damaged, it can still be replaced. Owing to the 3D printing technology, only the inner liner needs to be replaced, making the process cost-effective and convenient.

### 2.2. The Design of the PCB

The PCB was devised with an electronic design automation (EDA) software called EasyEDA. It was materialized according to the standard 4-layer PCB manufacturing and assembly protocol (Appendix A). Specifically, the environmental collection section of the system comprised three main components: the DHT11 temperature and humidity sensor module, the digital light intensity sensor module with built-in analog-to-digital converter (ADC), and the soil moisture sensor module utilizing analog data collection. The DHT11 sensor module consisted of a resistive humidity measuring element and a negative temperature coefficient (NTC) temperature measuring element. To collect real-time local air humidity and temperature, the Arduino Nano was connected to the sensor module through a circuit. The BH1750 light sensor module internally consisted of photodiodes, operational amplifiers, ADC acquisition, crystal oscillators, and BH1750 chips. The input optical signal was collected by ADC through the diode voltage through the photoelectric effect. The signal was then converted into a 16-bit binary number using a designed logic circuit and stored in the register within the BH1750 chip. The resistive soil moisture sensor module primarily consisted of a voltage comparator, a voltage divider circuit module, and electrodes. By connecting the electrodes to the soil, which acted as a variable resistor, the analog quantity was obtained through a series-connected voltage divider circuit and voltage comparator module. The original data were processed using the Arduino Nano. To optimize space utilization and facilitate data collection, a three-hole busbar was installed on the PCB to connect to the DHT11 temperature and humidity sensor, while a four-hole busbar was used for the BH1750 light sensor. For soil moisture collection, a voltage comparator and voltage divider module were integrated on the PCB connected to external electrodes. The Arduino Nano, featuring the ATmega168 processor core, was responsible for data processing, boasting high precision, fidelity, anti-interference performance, and low energy consumption. The execution aspect was primarily controlled by two JQC-3FF-S-Z relays, utilizing low current to manage high current operations, such as controlling LED fill lights and water pumps, respectively.

Data transmission and power modules are also necessary conditions for the sensory system. Wireless communication transmission with Android phones was achieved using the ESP8266 module, which included high speed cache memory to enhance overall system performance. The two microcontrollers can be interconnected via the TX/RX interface or the advanced high-performance bus (AHB) bridge interface of the central processor for signal exchange. The sensing system is powered by a rechargeable lithium-ion battery (5600 mAh 5 V). To facilitate power supply, the PCB board is equipped with an external power supply pin. The positive and negative electrode wires of the battery are connected to this pin, allowing it to provide the necessary power to the PCB board. The relevant codes were attached to Supplementary Materials.

### 2.3. Design of the Mobile Application

The mobile application (April-smart flowerpot) was developed by using the MIT App Inventor called App Inventor 2, which is based on visual block language. It is an online Android programming environment, different from traditional platforms based on Java or Kotlin languages. App Inventor is a tool used to develop Android smartphone programs, using building blocks for programming. In App Inventor 2, user interface development and program logic development are both visual and modular. Divided into different category modules, each module represents a different color and executes different instructions. The logical design of the software combines puzzle and building blocks, making it clear at a glance. Currently, the platform supports the logic and design of iPhone, Android, and iPad apps.

### 2.4. Acquisition of Soil Moisture Sensor Signals

Assemble the hardware system and flowerpot and connect the hardware system with a computer. First, insert the soil sensor into the soil to obtain the output value of soil data. The output value in the code written is voltage, ranging from 0 to 100% soil humidity, where one soil humidity value corresponds to one voltage output value. The soil humidity of 0 corresponds to a 5 V voltage, and for every 10% increase in humidity, the output voltage decreases by 0.5 V. The theoretical soil moisture is measured using the soil drying method. The tested sample is placed in a drying oven and dried to a constant weight at 105 °C, and then the soil moisture is calculated. This method can be used to determine whether the resistive soil moisture sensor is ready and calibrated. Soil moisture = (weight of sample soil and iron box before drying − weight of sample soil and iron box after drying)/(mass of sample soil and iron box before drying − mass of iron box) × 100%.

### 2.5. Acquisition of Light Intensity Sensor Signals

In the microcontroller, the data transmitted by the light sensor are collected. Firstly, place the light intensity tester in a fixed position and record the markings. Illuminate with a strong incandescent lamp to obtain a light intensity of 1000 Lux. Then, remove the tester and place the light sensor on the marked area. Use the same strong incandescent lamp to illuminate the data of the light sensor and collect the sample points. Repeat the above steps to change the light intensity to 2000 Lux, 3000 Lux, 4000 Lux, 5000 Lux, 6000 Lux, and collect the data transmitted by the sensor. This method can be used to verify and calibrate the light sensor system. Finally, the light intensity collected by the sensor corresponds one-to-one with that monitored by the light intensity tester.

### 2.6. Acquisition of Temperature and Humidity Sensor Signals

Use the DHT11 temperature and humidity sensor to measure indoor temperature and humidity. Measure at six time points: 12:30, 13:00, 13:30, 14:00, 14:30, and 15:00. Measure the output temperature and humidity values at each time point and compare them with the Xiaomi temperature and humidity measuring instrument (Xiaomi, Beijing, China) at the same time point.

### 2.7. Automatic and Remote Control

After powering on the PCB board, when the resistive soil moisture sensor detects that the soil humidity is below 30%, control the relay switch to turn on the water pump to achieve automatic watering; when the humidity reaches 60%, control the relay to shut down the water pump. When the light sensor detects that the light intensity is less than 5000 Lux, the relay is controlled to turn on the fill light to supplement light for the plant. When the light intensity exceeds 5000 Lux, the fill light is turned off.

## 3. Results and Discussion

### 3.1. Structure

**Figure 2:**
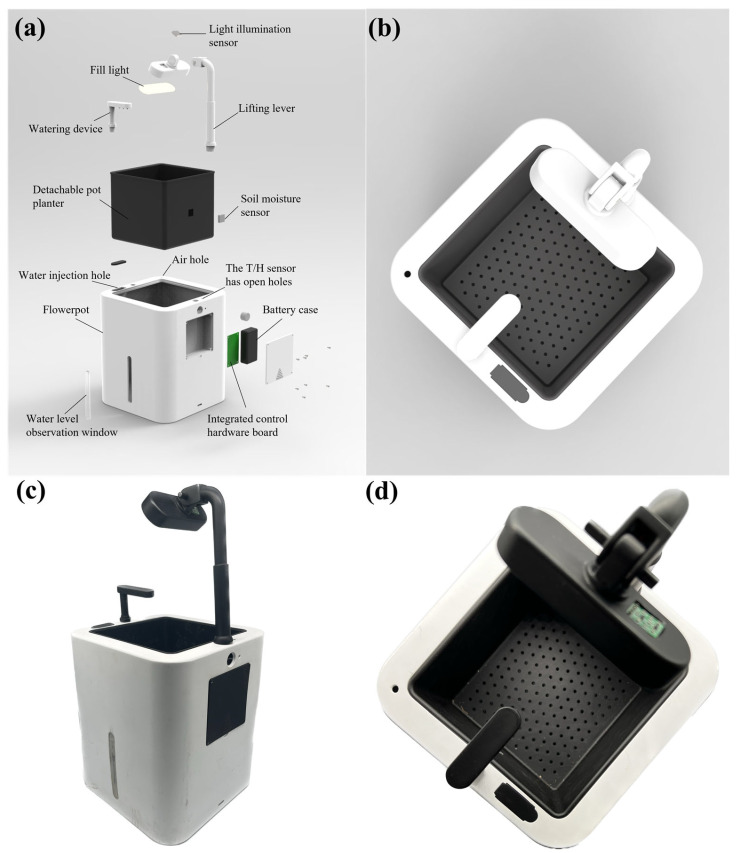
Schematic and physical figures of the flowerpot structure (**a**) Exploded figure. (**b**) Top view of the flowerpot. (**c**,**d**) Physical images of the flowerpot.

For the vital feature of smart flowerpot products—to ensure that the pot has more space to store water and soil, the weight and size of the hardware system should be reduced as much as possible while the functions of the smart flowerpot are made more complete. The core control chip of this smart flower pot is the Arduino nano [48]. The control circuit is composed of a DH11 humidity sensor, temperature sensor, and resistive soil moisture sensor, a BH1750 strong light sensor, a high light LED fill light circuit, and an automatic watering control circuit. The whole flowerpot system is composed of an extendable support replenishing light, a watering device, and other additional components. The structure of the flowerpot is shown in Figure 2.

The design of the shell is a square flowerpot with a length and width of 18 cm and a height of 21 cm. Because when the sides of a square are equal to the diameter of a circle, the area of a square is larger, a cuboid pot can store more water at the same height. An auxiliary light-emitting apparatus has been engineered with the capacity for angle adjustment, thereby offering dynamic control over light intensity. This component incorporates a lampshade and a support rod, interconnected by means of pins, which facilitate the unrestricted angular adjustment of the lampshade, enabling an effective modulation of light intensity. In addition, we reserved space on the lampshade to place the light sensor. The light hits the plant at roughly the same angle as it hits the lampshade, so the lampshade is the best place to put the light sensor. At the same time, we also designed a transparent water level observation window to observe whether the watering system in the pot is storing enough water. The watering device of the automatic watering system was designed in the shape of a shower. The use of sprinkler watering can save more water resources at the same time to spray the same area of soil, uniformly wetting the soil quickly. The plant cultivator was designed to be removable. Detachable flowerpots allow users to change the plants they want to grow. Users are afforded the convenience of directly substituting the plant cultivator, thereby negating the need for the extraction and subsequent replanting of the original flora. Furthermore, the design facilitates expedient and effortless soil aeration through the removal of the plant cultivation box, thus enhancing the system’s overall user-friendliness and operational efficiency. In addition, the bottom of the pot was designed with a hole to ensure that there is a certain amount of oxygen in the soil, to prevent plants from excess watering and rotting the roots, and to support drainage and ventilation.

### 3.2. Working Principle

**Figure 3:**
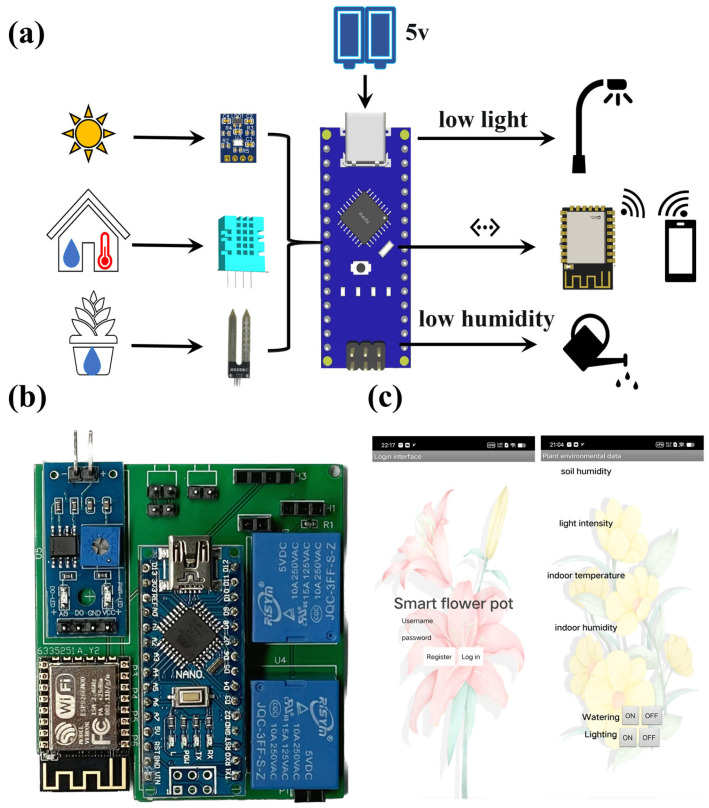
Hardware and software of the smart flowerpot system. (**a**) The block diagram of the system. (**b**) The photo of PCB. (**c**) The homepage of the mobile application named April-smart flowerpot.

The smart flowerpot measures the parameters of the plant growth environment, such as temperature, humidity, soil moisture, and light intensity, through the circuit of the DHT11 temperature sensor, resistive soil moisture sensor, and the BH1750 light sensor. The control circuit controlled the relay when the plant growth parameters measured by the resistive soil moisture sensor and the BH1750 light sensor were not in the normal range. Thus, it can control the water pump and the LED light, such as automatic watering and light filling. The smart flowerpot was controlled by a mobile phone application using Wi-Fi (ESP8266 Wi-Fi) [48]. Users can query plant growth environment parameters and control watering and filling light. The system block diagram and the circuit diagram of the designed smart flowerpot system are illustrated in Figure 3a and Appendix A, respectively.

The size of the entire acquisition circuit is 60 mm × 53 mm, which integrates the acquisition and control circuits, as shown in Figure 3b. The Arduino nano is placed in the middle and connected to the busbar with a row pin for easy disassembly of the microcontroller. Place the ESP8266 Wi-Fi module in the bottom left corner and empty the bottom left corner of the board by 10 mm × 30 mm. Because the PCB board design adopts a copper laying design for more stable conductivity and system stability, this design prevents a certain amount of interference caused by the Wi-Fi module of the ESP8266 coming into contact with copper. The two relays were placed on the upper right side of the PCB to control the fill light. The two-hole busbar next to the upper relay is the fill light connection port. The lower relay controls the water pump, and the two-hole busbar next to the lower relay is the water pump connection port. Place the soil sensor at the top left for convenience in placing the sensor in the soil. In addition, the printed circuit board was also designed with external power supply pins, and an external circuit power supply port was designed at the top for convenience in powering on the board. In addition, the top four-hole busbar is the light sensor placement port, while the three-hole busbar is the temperature and humidity sensor placement port. Connecting a 4.7 KΩ resistor is more accurate for reading the value. The board weighs a total of 40 g and integrates an Arduino Nano, three sensors, and an ESP8266 Wi-Fi module. The system hardware wiring diagram is illustrated in Figure S2. The cost of the integrated smart flowerpot is $21.19 (Table S2).

The sensing system is powered by a rechargeable lithium-ion battery (5600 mah 5 V, Asia Electronics), which is small in size (70 mm × 37 mm × 19 mm) and takes up less space, reducing the weight of the smart flowerpot and improving its suitability. The battery capacity display is 25%, 50%, 75%, and 100%, respectively. When the battery is out of power, it can be taken off separately to charge the battery. The main control chip of the hardware control system is the Arduino nano [49]. The size of the nano is very small, and the space of the nano is much smaller with the same function. The size of the Arduino nano is only 45 × 18 mm; it is lightweight and simple and can be directly designed on printed circuit boards. The processor core is ATmega328, which has 14 digital input/output ports, including 6 for PWM output and 8 for analog input, a 16 MHz crystal oscillator, a mini-B USB port, an ICSP header, and a reset button. Therefore, using the nano as the main control chip can reduce the area of the integrated circuit board. The Arduino nano chip features high accuracy, high fidelity, high anti-interference performance, and low power consumption [50]. The light sensor uses a BH1750 strong light acquisition sensor. The stronger the light entering the light window, the greater the photocurrent and the greater the voltage it outputs. Therefore, the light intensity can be judged by the voltage. The sensor has a measuring range of 0 to 60,000 Lux, which is sufficient for the light intensity required by smart flowerpot hardware. Most house potted plants grow at a light intensity of 3000 Lux to 10,000 Lux [51]. When the BH1750 detects that the light intensity is less than 5000 Lux, the relay will be controlled to turn on the fill light. The soil moisture sensor of the smart flowerpot is a resistive soil moisture sensor. The sensor is composed of two electrodes, which are used to connect the variable resistance of the soil so that it can pass current [52]. It can judge the degree of dry and wet soil according to the change of output voltage. Suitable soil moisture for most houseplants is 20% to 60% [53]. When the soil moisture is less than 30%, the relay is controlled to turn on the water pump. When the soil moisture reaches 60%, the pump stops watering. For data transmission, the Arduino nano collects the data of the train sensor and sends them to the mobile application through the ESP8266 Wi-Fi module [54].

### 3.3. Design of the Mobile Application

A mobile application has been developed for Android smartphones that communicates with the Smart flowerpot hardware system. The application was built on the App inventor 2 platform (MIT App Inventor) [55]. The platform is based on a visual language. All the user must do is coding for the required assembly program, and this makes it easy to understand programming. The application is named April-smart flowerpot. Figure 3c and Figure S3 show the April-smart flowerpot. As Figure S3a shows, the first screen is a login screen, where new users need to register their accounts, while old users only need to log in to their registered accounts to access the second screen. The second interface which is illustrated in Figure S3b demonstrates real-time data on the growing environment of the plant in the pot. The blank space after indoor temperature indicates the air temperature collected in real time by the temperature and humidity sensor (DHT11), and the blank space after indoor humidity indicates the air humidity collected in real time by the temperature and humidity sensor (DHT11). The blank space behind the light intensity is the light intensity collected by the light illumination sensor (BH1750) in real time, and the blank space behind the soil moisture is the potting soil moisture collected by the resistive soil moisture sensor in real time. Users can view real-time data of the plant environment in time. In addition, there are water and fill light buttons, water, and fill light for plants in pots. When the plant condition is up to standard, users can choose to stop watering or fill the light.

### 3.4. Calibration of the Sensors

**Figure 4:**
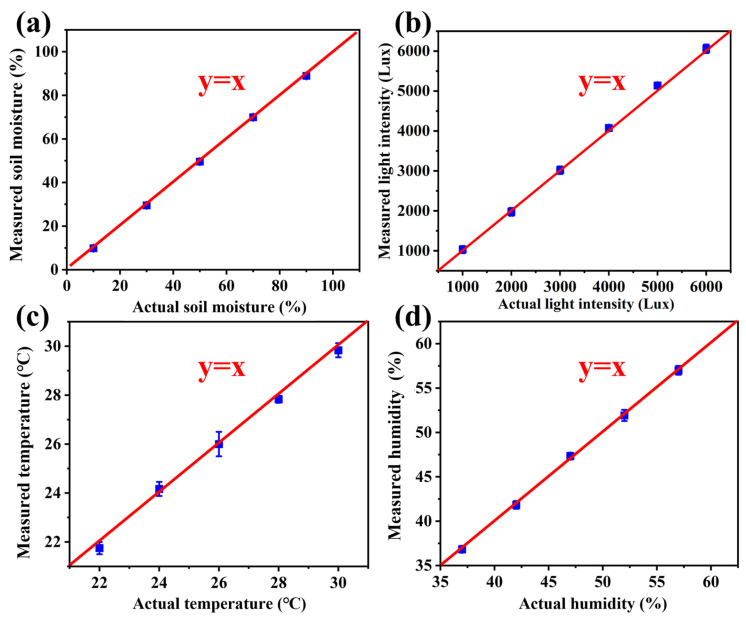
Calibration of actual soil humidity, light intensity, and temperature using the multi-sensor-based smart flowerpot. (**a**) Actual soil moisture calibrated with measured soil moisture (**b**) Light intensity measured with the light sensor compared with actual light intensity. (**c**,**d**) Actual indoor temperature and humidity compared with the data measured.

The smart flowerpot was placed on a table for calibration of the sensors. For the resistive soil moisture sensor module, the voltage signal output can be received from its monitoring of soil moisture. Hence, the detected data can be obtained. After using the soil drying method, the actual soil moisture data were obtained. As shown in Figure 4a, the detected soil moisture measurement calculated from the received voltage signal was calibrated with actual soil moisture data. The function between the actual and measured soil moisture is presented in Equation (1).

y = x,(1)

Light is vital for a plant’s growth. As shown in Figure 4b, the actual and measured light intensities are equal. It is evident that the light sensor measures precisely. The two output feature functions are linear. Hence, it is stable when monitoring the real-time data. Similarly, as for temperature and humidity, the data it monitored compared with that of the actual environment (Figure 4c,d). The detected values were basically the same as the actual values, so the entire sensor system data were accurate and reliable.

### 3.5. Practical Application

**Figure 5:**
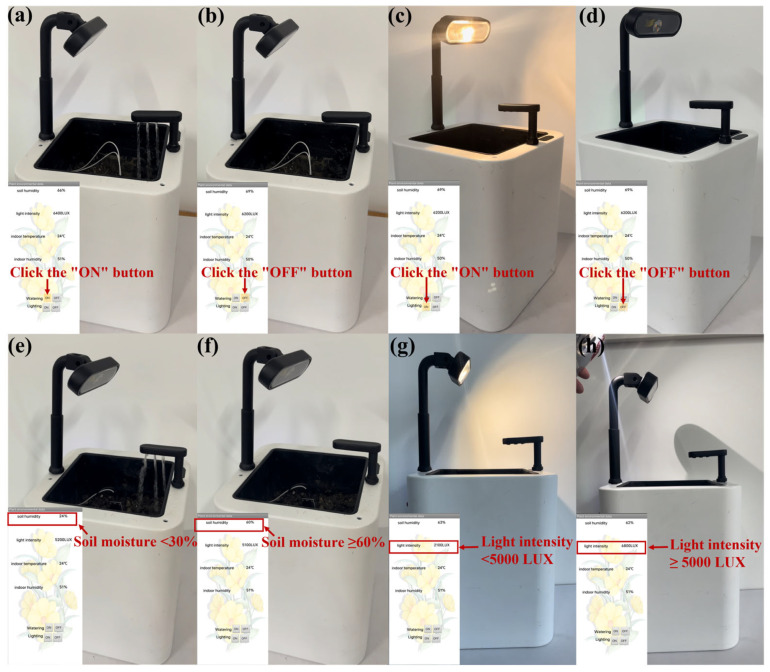
The smart flowerpot automatic control and manual control of filling light and watering. (**a**) Manual control of turning on the water pump via April-smart flowerpot by clicking the “ON” button. (**b**) Manual control of turning off the water pump via April-smart flowerpot by clicking the “OFF” button. (**c**) Manual control of turning on the fill light via April-smart flowerpot by clicking the “ON” button. (**d**) Manual control of turning down the fill light via April-smart flowerpot by clicking the “OFF” button. (**e**) Turning on the water pump to water automatically when soil moisture is 24% (lower than 30%). (**f**) Stopping watering when soil moisture is 60% (greater than or equal to 60%). (**g**) Turning on the fill light when light intensity is 2100 Lux (lower than 5000 Lux). (**h**) Turning off the fill light when light intensity is 6800 Lux (greater than or equal to 5000 Lux).

After monitoring the real environment, the smart flowerpot was used in a practical application. First, the wireless control via the designed application is also significant for everyday usage scenarios. In Video S1 and Figure 5a, users can control the fill light and water pump through the application. Users clicked the “ON” button to control the relay to turn on the water pump to realize the function of watering. After clicking the “OFF” command, the mobile phone software sent “OFF” instructions to the Bamfa Cloud, which sent instructions to the ESP8266 module through Wi-Fi, and the ESP8266 module realized the information interaction with the single-chip microcomputer to control the relay to turn off the water pump and stop watering (Figure 5b). As shown in Video S2 and Figure 5c, when users clicked the “ON” button, the mobile phone software sent “ON” instructions to the Bamfa Cloud, the Bamfa Cloud sent instructions to the ESP8266 module through Wi-Fi, and the ESP8266 module interacted with the single-chip microcomputer to control the relay to turn on the fill light to realize the function of filling light. Then, the user clicked the “OFF” button, and the relay was controlled to turn off the fill light (Figure 5d).

Next comes the automatic control of watering and filling light. As shown in Video S3 and Figure 5e, the resistive soil moisture sensor measured that the soil moisture was lower than the standard of the plant’s growth (30%), and the relay controlling the water pump was turned on to pump water. The water pump was stopped by the relay until the soil moisture sensor monitored that the soil moisture was greater than or equal to 60% (Figure 5f). As for filling light automatically, the light sensor detected when the environment’s light intensity was not adequate for the plant’s growth (lower than 5000 Lux). Then, the relay controlled the fill light to turn it on (Figure 5g). In Video S4 and Figure 5h, at first the fill light turned on due to the poor light intensity. As we held the flashlight close to the flowerpot to simulate an increase in ambient light intensity, the light sensor detected an increase in ambient light intensity, and the relay controlled the fill light to stop filling the light.

**Figure 6:**
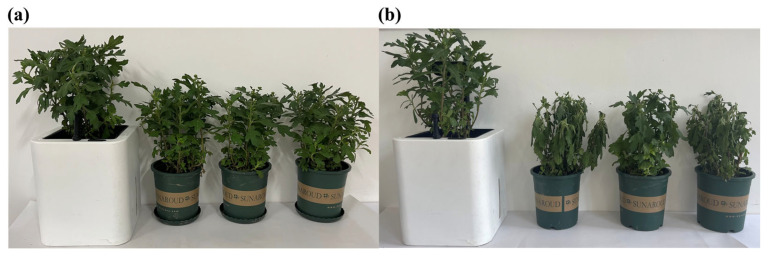
The actual cultivation of chrysanthemums in a comparison between the smart flowerpot and ordinary flowerpots. (**a**) The first day of actual cultivation of the four chrysanthemums. (**b**) After 7 days the actual cultivation of the four chrysanthemums.

We simulated a scenario in which the user was on a business trip for 7 days and neglected to care for the chrysanthemums. We conducted a comparison between the chrysanthemums cultivated in the smart flowerpot and ordinary flowerpots (Figure 6). Figure 6a illustrates the view of the first day that the user went on a business trip. During the 7 days, the four flowerpots were placed on the balcony, and the smart flowerpot could automatically water and fill light for the chrysanthemum, but the chrysanthemums in the ordinary flowerpots were not cared for. The average temperature of the 7 days was about 30 degrees Celsius. Figure 6b demonstrated the view of the plants after 7 days. During the 7 days, the chrysanthemums in the ordinary flowerpots lacked supplementary water. In addition, the filling light was also missing. It is obvious that the plants in the ordinary flowerpots were languid.

## 4. Conclusions

In summary, we have illustrated a personalized and smart flowerpot enabled by 3D printing and cloud technology for ornamental horticulture for monitoring environmental parameters in real-time and cultivating plants by filling light and watering automatically. The smart flowerpot mainly contains a battery, resistive soil moisture sensor, light intensity sensor, fill light, watering device, flowerpot, integrated control hardware board, and an application named April-smart flowerpot. As for the calibration of sensors, they measure parameters of the environment continuously and accurately and display real-time data in the application interface. Also, it can control the fill light and water pump automatically to supplement light and water if the microcontroller accepts the abnormal data from sensors. In addition, users can control it through the application. The demonstrated smart flowerpot promotes the development of smart devices and their applications in smart homes, ornamental horticulture, and so on.

## Figures and Tables

**Figure A1:**
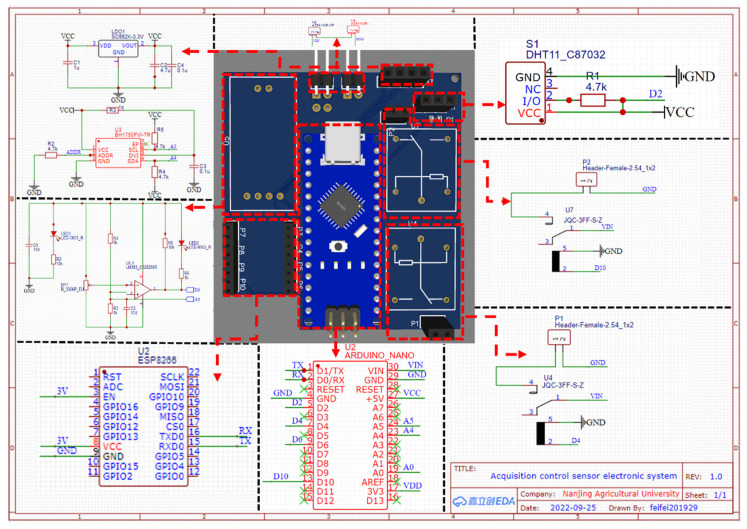
The circuit diagram of the system of the electronic sensors.

## Data Availability

Data will be made available on request.

## Supplementary Materials

The following supporting information can be downloaded at: https://www.mdpi.com/article/10.3390/s23136116/s1, Table S1: Flowerpots’ function matrix; Table S2: Bill of materials for the electronic sensory system; Figure S1: 3D printing machine; Figure S2: Hardware system wiring diagram; Figure S3: The software application interface. (a) Login interface (b) Plant data display interface; Code S1: Arduino controls the operation of the fill light; Code S2: Arduino controls the operation of the water pump; Code S3: Arduino collects air temperature and humidity; Code S4: Mobile phone control for watering and lighting; Code S5: Real-time data acquisition of plant growth environment using mobile phones; Video S1: Users manually control watering on the application interface; Video S2: Users manually control fill light on the application interface; Video S3: The smart flowerpot’s automatic control of watering; Video S4: The smart flowerpot’s automatic control of supplemental light.
