# Supplementary material for: A Personalized and Smart Flowerpot Enabled by 3D Printing and Cloud Technology for Ornamental Horticulture
Source: Sensors (Basel). 2023 Jul 3;23(13):6116. doi: 10.3390/s23136116 (PMC10346579; doi:10.3390/s23136116)
Supplement: Supplementary file 1 [file sensors-23-06116-s001.zip › sensors-2472819-supplementary.pdf]

Supplementary Materials

A personalized and smart flowerpot enabled by 3D printing and cloud technology for ornamental horticulture

Yecheng Li [a,1], Jiaxing Luo [a,1], Daosheng Wu [a], and Cheng Zhang [a,*]

[a]College of Engineering, Nanjing Agricultural University, Nanjing 210031, China.

[1]These authors contributed equally: Yecheng Li, Jiaxing Luo
*Correspondence: Cheng Zhang (zhangcheng@njau.edu.cn)

Table of Contents

1.Table S1. Flowerpots' function matrix.

2.Table S2. Bill of materials for the electronic sensory system.

3. Figure S1. 3D printing machine.

4. Figure S2. Hardware system wiring diagram.

5. Figure S3. The software application interface. (a) Login interface (b) Plant data display interface.

6. Code S1. Arduino controls the operation of the fill light.

7. Code S2. Arduino controls the operation of the water pump.

8. Code S3. Arduino collects air temperature and humidity.

9. Code S4. Mobile phone control for watering and lighting.

10. Code S5. Real-time data acquisition of plant growth environment using mobile phones.

11. Video S1. Users manually control watering on the application interface.

12. Video S2. Users manually control fill light on the application interface.

13. Video S3. The smart flowerpot's automatic control of watering.

14. Video S4. The smart flowerpot's automatic control of supplemental light.

Formatted: Not Highlight

Formatted: Not Highlight

Table S1. Flowerpots' function matrix.

Automatic watering	Automatic filling light	Real-time data transmission	mobile APP	Light intensity monitoring	Soil moisture monitoring	Temperature and humidity monitoring	Mobile control	Flowerpot materials	Ref.
yes	yes	yes	no	no	yes	yes	no	no	[36]
yes	no	yes	yes	yes	yes	yes	no	no	[37]
yes	yes	yes	yes	no	yes	yes	no	no	[38]
yes	yes	no	no	yes	yes	yes	no	no	[39]
no	no	yes	no	no	yes	no	no	no	[40]
yes	no	yes	no	no	yes	no	no	3D Printing	[41]

Table S2. Bill of materials for the electronic sensory system.

Components	Functions	Quantity	Approximate Cost ($)	Manufacturer or Supplier
Arduino nano	Control	1	3.71	Xin Microelectronics Technology
DHT11 Temperature and humidity sensor	Indoor temperature and humidity	1	0.33	Uxin Electronic Technology
BH1750 Light sensor	Light intensity	1	1.45	Weiping trade
Soil moisture sensor	Soil moisture	1	0.44	Specific micro semiconductor
JQC-3FF-005-S-Z relay	Control switch	2	0.82	Specific micro semiconductor
5Vflling lamp	Fill light	1	0.73	Shitong light source
Water pump	water	1	0.89	Specific micro semiconductor
Water pipe	water	1	0.73	Specific micro semiconductor
ESP8266	wi-Fi	1	2.18	Specific micro semiconductor
1000ohm resistance	circuit	1	0.1	Specific micro semiconductor
Three-hole bushing	circuit	1	0.1	Specific micro semiconductor
Four-hole bushing	circuit	1	0.1	Specific micro semiconductor
Two-hole bushing	circuit	2	0.1	Specific micro semiconductor
5600 mAh 5 V Lithium Battery	Power supply	1	5.46	Middle Road Energy Technology
53mm×60mm Acquisition circuit board	Power supply	1	0.57	Calicron
PLA	3D printing		2.66	Lanbo
Total cost			21.19	

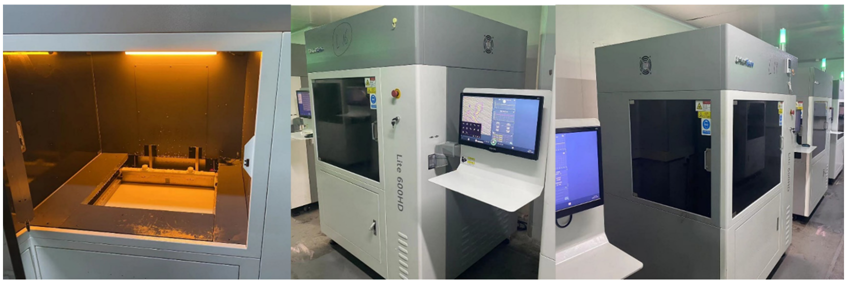

Figure S1 3D printing machine.

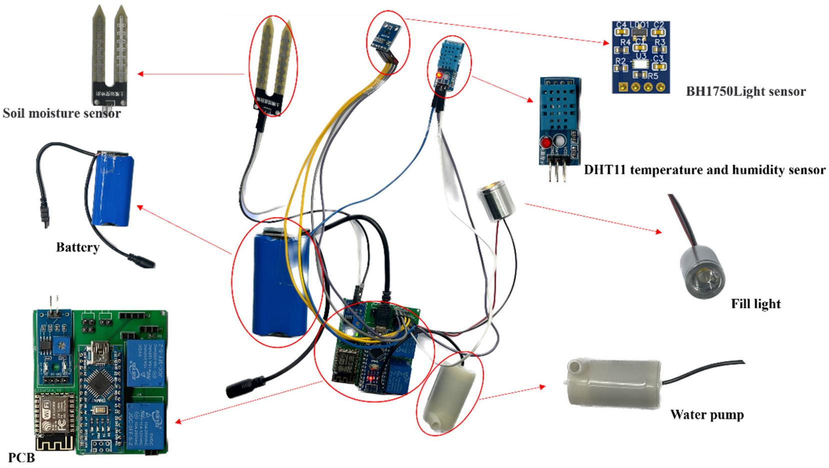

Figure S2~~3~~ Hardware system wiring diagram.

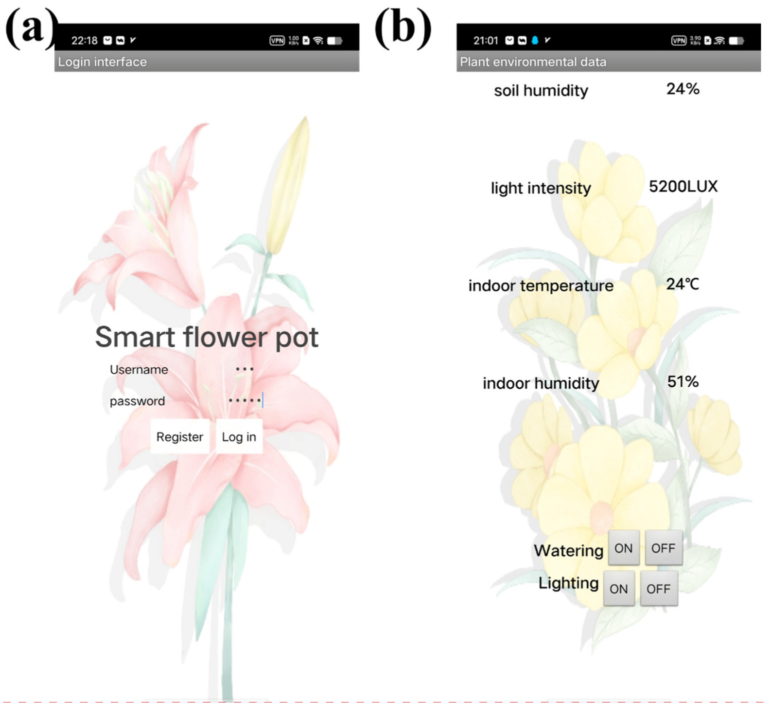

Figure S3 The software application interface. (a) Login interface (b) Plant data display interface.

Code S1 Arduino controls the operation of the fill light.

```
int ledPin=10;

#include <Wire.h>

#include <math.h>

#define     BH1750_ONE_H2     0x21

byte buff[2];

void setup()

{

Wire.begin();

Serial.begin(9600);
```

Formatted: Not Highlight

```
pinMode(ledPin,OUTPUT);
}
void loop()
{
Serial.println( BH1750() );
if(BH1750()<=5000)
{
   digitalWrite(ledPin,HIGH);
}
else
{
digitalWrite(ledPin,LOW);
}
}
double BH1750()
{
       int i=0;
       double val=0;
       Wire.beginTransmission(BH1750address);
       Wire.write(0x21);//1lx reolution 120ms
       Wire.endTransmission();
       delay(200);
```

```
    Wire.beginTransmission(BH1750address);
    Wire.requestFrom(BH1750address, 2);
    while(Wire.available())
    {
      buff[i] = Wire.read();
      i++;
    }
    Wire.endTransmission();
    if(2==i)
    {
      val=((buff[0]<<8)|buff[1])*0.5/1.2;
    }
     return val;
}
```

Code S2 Arduino controls the operation of the water pump.

```
int sensorpin = 6;
float pumpin = 4;
int moisture1;
int moisture3;
int sensorpin2 = A0;
void setup() {
```

```
    Serial.begin(9600);
    pinMode(sensorpin,INPUT);
    pinMode(sensorpin2,INPUT);
    pinMode(pumpin,OUTPUT);
    digitalWrite(pumpin,LOW); }
void loop() {
    moisture1 = digitalRead(sensorpin);
    int moisture2=digitalRead(pumpin);
    moisture3=analogRead(sensorpin2);
    Serial.print("moisture3:");
    Serial.println(moisture3);
  if(moisture3>580)
  {
    Serial.print(moisture3);
    digitalWrite(pumpin,HIGH);
    delay(2000);
    digitalWrite(pumpin,LOW);
      delay(5000);
  }
   else if(moisture3>=320&&moisture3<=580)
     {
      Serial.print(moisture3);
```

```
            digitalWrite(pumpin,LOW);
        }
else
        {
            Serial.print(moisture3);
            digitalWrite(pumpin,LOW);
        }
      delay(1000);
      //delay(18000);
}
```

Code S3 Arduino collects air temperature and humidity.

```
#include <DFRobot_DHT11.h>
DFRobot_DHT11 DHT;
#define DHT11_PIN 2
void setup(){
    Serial.begin(9600);
}
void loop(){
    DHT.read(DHT11_PIN);
    Serial.print("temperatue:");
    Serial.print(DHT.temperature);
```

```
    Serial.print("    humidity:");
    Serial.println(DHT.humidity+14);
    delay(1000);
}
```

Code S4 Mobile phone control for watering and lighting.

```
#include <ESP8266WiFi.h>
#define TCP_SERVER_ADDR "bemfa.com"
#define TCP_SERVER_PORT "…….."
#define DEFAULT_STASSID    "………."
#define DEFAULT_STAPSW      "…….
String UID = "…….";
String TOPIC =      "…….";
String TOPIC =      "…….";
const int LED_Pin = D10;
const in watering Pin = D4;
#define MAX_PACKETSIZE 512
#define KEEPALIVEATIME 60*1000
WiFiClient TCPclient;
String TcpClient_Buff = "";
unsigned int TcpClient_BuffIndex = 0;
unsigned long TcpClient_preTick = 0;
```

```
unsigned long preHeartTick = 0;
unsigned long preTCPStartTick = 0;
bool preTCPConnected = false;
void doWiFiTick();
void startSTA();
void doTCPClientTick();
void startTCPClient();
void sendtoTCPServer(String p);
void turnOnLed();
void turnOffLed();
void turnOnwatering();
void turnOffwatering();
void sendtoTCPServer(String p){
  if (!TCPclient.connected())
  {
    Serial.println("Client is not readly");
    return;
  }
  TCPclient.print(p);
  Serial.println("[Send to TCPServer]:String");
  Serial.println(p);
  preHeartTick = millis();
```

```
}
void startTCPClient(){
  if(TCPclient.connect(TCP_SERVER_ADDR, atoi(TCP_SERVER_PORT))){
    Serial.print("\nConnected to server:");
    Serial.printf("%s:%d\r\n",TCP_SERVER_ADDR,atoi(TCP_SERVER_PORT));
    String tcpTemp="";
    tcpTemp = "cmd=1&uid="+UID+"&topic="+TOPIC+"\r\n";
    sendtoTCPServer(tcpTemp);
    tcpTemp="";
    preTCPConnected = true;
    TCPclient.setNoDelay(true);
  }
  else{
    Serial.print("Failed connected to server:");
    Serial.println(TCP_SERVER_ADDR);
    TCPclient.stop();
    preTCPConnected = false;
  }
  preTCPStartTick = millis();
}
void doTCPClientTick(){
   if(WiFi.status() != WL_CONNECTED) return;
```

```
if (!TCPclient.connected()) {
if(preTCPConnected == true){
    preTCPConnected = false;
    preTCPStartTick = millis();
    Serial.println();
    Serial.println("TCP Client disconnected.");
    TCPclient.stop();
}
else
{
    if (TCPclient.available()) {
        char c =TCPclient.read();
        TcpClient_Buff +=c;
        TcpClient_BuffIndex++;
        TcpClient_preTick = millis();
        if(TcpClient_BuffIndex>=MAX_PACKETSIZE - 1){
            TcpClient_BuffIndex = MAX_PACKETSIZE-2;
            TcpClient_preTick = TcpClient_preTick - 200;
        }
    }
    if(millis() - preHeartTick >= KEEPALIVEATIME){
        preHeartTick = millis();
```

```
            Serial.println("--Keep alive:");
            sendtoTCPServer("cmd=0&msg=keep\r\n");
        }
    }
    if((TcpClient_Buff.length() >= 1) && (millis() - TcpClient_preTick>=200))
    {//data ready
        TCPclient.flush();
        Serial.print("Rev string: ");
        TcpClient_Buff.trim();
        Serial.println(TcpClient_Buff); /
        String getTopic = "";
        String getMsg = "";
String getTopic 2= "";
        String getMsg 2= "";
        if(TcpClient_Buff.length() > 15){ String TcpClient_Buff = "";
                cmd=2&uid=xxx&topic=light002&msg=off
                  int topicIndex = TcpClient_Buff.indexOf("&topic=")+7;
                  int msgIndex = TcpClient_Buff.indexOf("&msg=");
                  getTopic = TcpClient_Buff.substring(topicIndex,msgIndex);/
                  getMsg = TcpClient_Buff.substring(msgIndex+5);
                  Serial.print("topic:------");
                  Serial.println(getTopic);
```

```
                    Serial.print("msg:--------");
                    Serial.println(getMsg);
      }
      if(getMsg    == "on"){
          turnOnLed();
      }else if(getMsg == "off"){
            turnOffLed();
        }
if(getMsg2    == "on"){
          turnOnwatering();
      }else if(getMsg == "off"){
            turnOffwatering();
        }
      TcpClient_Buff="";
      TcpClient_BuffIndex = 0;
    }
}
void startSTA(){
    WiFi.disconnect();
    WiFi.mode(WIFI_STA);
    WiFi.begin(DEFAULT_STASSID, DEFAULT_STAPSW);
}
```

Code S5 Real-time data acquisition of plant growth environment using mobile phones.

```
void startTCPClient(){
  if(TCPclient.connect(TCP_SERVER_ADDR, atoi(TCP_SERVER_PORT))){
    Serial.print("\nConnected to server:");
    Serial.printf("%s:%d\r\n",TCP_SERVER_ADDR,atoi(TCP_SERVER_PORT));
    preTCPConnected = true;
    preHeartTick = millis();
    TCPclient.setNoDelay(true);
  }
  else{
    Serial.print("Failed connected to server:");
    Serial.println(TCP_SERVER_ADDR);
    TCPclient.stop();
    preTCPConnected = false;
  }
  preTCPStartTick = millis();
}
void doTCPClientTick(){
   if(WiFi.status() != WL_CONNECTED) return;
  if (!TCPclient.connected()) {
  if(preTCPConnected == true){
```

```
    preTCPConnected = false;
    preTCPStartTick = millis();
    Serial.println();
    Serial.println("TCP Client disconnected.");
    TCPclient.stop();
}
else if(millis() - preTCPStartTick > 1*1000)
    startTCPClient();
}
else
{
    if (TCPclient.available()) {
        char c =TCPclient.read();
        TcpClient_Buff +=c;
        TcpClient_BuffIndex++;
        TcpClient_preTick = millis();
        if(TcpClient_BuffIndex>=MAX_PACKETSIZE - 1){
            TcpClient_BuffIndex = MAX_PACKETSIZE-2;
            TcpClient_preTick = TcpClient_preTick - 200;
        }
        preHeartTick = millis();
    }
```

```
        if(millis() - preHeartTick >= upDataTime){
            preHeartTick = millis();
            int data1 = A0
            float data2 =A4;
            unsigned int data3 =D2;
            int data4=D2;
            String upstr = "";
            upstr
"cmd=2&uid="+UID+"&topic="+TOPIC+"&msg=#"+data1+"#"+data2+"#"+data3+"
#"+data4+"#"
            sendtoTCPServer(upstr);
            upstr = "";
        }
    }
    if((TcpClient_Buff.length() >= 1) && (millis() - TcpClient_preTick>=200))
    {//data ready
        TCPclient.flush();
        Serial.println("Buff");
        Serial.println(TcpClient_Buff);
      TcpClient_Buff="";
      TcpClient_BuffIndex = 0;
    }
```

```
}
void startSTA(){
  WiFi.disconnect();
  WiFi.mode(WIFI_STA);
  WiFi.begin(DEFAULT_STASSID, DEFAULT_STAPSW);
}
```
